# Prognostic implications of obstructive sleep apnea in patients with acute coronary syndrome stratified by homocysteine level: a prospective cohort study

**DOI:** 10.1186/s12931-023-02627-8

**Published:** 2023-12-14

**Authors:** Xiuhuan Chen, Lei Zhen, Hui Ai, Bin Que, Jingyao Fan, Xiao Wang, Yan Yan, Siyi Li, Zekun Zhang, Yun Zhou, Wei Gong, Shaoping Nie

**Affiliations:** 1 Center for Coronary Artery Disease, Division of Cardiology, Beijing Anzhen Hospital, Capital Medical University, 2 Anzhen Road, Chaoyang District, Beijing, 100029 China; 2 National Clinical Research Center for Cardiovascular Diseases, Beijing, China; 3 Beijing Institute of Heart, Lung, and Blood Vessel Diseases, Beijing, China

**Keywords:** Acute coronary syndrome, Obstructive sleep apnea, Homocysteine

## Abstract

**Background:**

Sporadic studies have examined the impact of OSA on ACS patients by homocysteine (Hcy) level. This study attempted to comprehensively evaluate the effects of the interaction between Hcy and OSA on long-term cardiovascular outcomes in ACS patients.

**Methods:**

In this prospective, large-scale cohort study, 2160 patients admitted for ACS were recruited to undergo overnight sleep monitoring. OSA was diagnosed when apnea–hypopnea index ≥ 15 events/h. Patients with normohomocysteinemia (NHcy) were defined as having serum Hcy ≤ 15 μmol/L, and the others had hyperhomocysteinemia (HHcy). The primary endpoint was major adverse cerebrocardiovascular event (MACCE), a composite of cardiovascular death, myocardial infarction, stroke, ischemia-driven revascularization and hospitalization for unstable angina and heart failure.

**Results:**

A total of 1553 eligible ACS patients (average age: 56.3 ± 10.5 years) were enrolled, among which 819 (52.7%) had OSA, and 988 (63.6%) were with NHcy. OSA did not significantly affect the level of Hcy. During a median follow-up of 2.9 (1.6, 3.5) years, after adjustment for clinical confounders, OSA was associated with increased risk for MACCE occurrence versus non-OSA ones in ACS patients with NHcy (adjusted hazard ratio [HR] = 1.36, 95% confidence interval [CI] 1.02–1.83, *P* = 0.039), but not in those with HHcy (adjusted HR = 0.92, 95%CI 0.62–1.36, *P* = 0.668). There was an absence of interaction between homocysteine level and OSA in relation to MACCE (interaction *P* = 0.106).

**Conclusions:**

OSA was independently associated with worse prognosis in ACS patients with NHcy. Our study emphasized the necessity to identify potential presence of OSA in such a population.

*Trial registration*: ClinicalTrials.gov; Number: NCT03362385; URL: www.clinicaltrials.gov.

**Supplementary Information:**

The online version contains supplementary material available at 10.1186/s12931-023-02627-8.

## Introduction

Obstructive sleep apnea (OSA), characterized by repetitive collapse of upper airway followed by snoring, intermittent hypoxemia, sleep fragmentation and autonomic oscillations, has been recognized as a serious global health burden in consideration of the prevalence and the detrimental impacts on neurocognition and cardiovascular systems [1, 2]. OSA is prevalent in general population that affects ~ 34% and 17% of men and women, respectively [3], which could remarkably increase to 50–65% in patients with established acute coronary syndrome (ACS) [4–7], with respect to the implications of OSA in both etiology and progression of ACS [8, 9]. Based on this notion, accumulating studies from our group and others have demonstrated that OSA contributes as an important risk factors to the occurrence of cardiovascular events in ACS patients during long-term follow-up [4, 5, 10]. Although the underlying mechanisms are intricate and obscure, concomitant metabolic disorders (e.g., hyperglycemia, dyslipidemia and hyperuricemia) function as one of the linchpins of this association [11–13], among which homocysteine (Hcy) is a potential risk factor [14, 15].

Hcy, a sulfhydryl-containing amino acid, is an intermediate product in the metabolism of methionine [16]. Elevated serum Hcy is involved in pathogenesis and progression of ACS [16–18]. Additionally, several investigators have found the correlation between OSA and Hcy that serum Hcy level increased with the severity of OSA [19, 20]. However, rigorously controlled comparative studies unraveled that OSA per se appeared not to promote the excess of Hcy [14, 21] but otherwise advanced age, obesity, renal dysfunction, thyroid diseases and various drugs accounted more for [15, 22], yielding the hypothesis that variance in Hcy level among OSA patients might be ascribed to distinct risk factor profiles concomitant with OSA [15]. From this perspective, serum Hcy could act as a biomarker reflecting the coexistence of OSA and metabolic disorders. In addition, it was also notable that Hcy and OSA shared analogous pathophysiological pathways in coronary atherogenesis, comprising vascular endothelial dysfunction, platelet aggregation, smooth muscle cell proliferation, oxidative stress, endothelia-leukocyte interaction and inflammatory infiltration [15, 23], contributing synergistically to the development of new-onset hypertension, increased episode of cardiovascular events and higher mortality during long-term follow-up in patients without prior ACS [23, 24].

Nevertheless, to our knowledge, effects of this interaction between OSA and Hcy on the long-term cardiovascular outcomes in patients with established ACS have not been previously evaluated. Therefore, based on a large-scale prospective cohort, we executed current study to investigate the chronic impact exerted by OSA on ACS patients in relation to Hcy level.

## Methods

### Study population

The OSA-ACS project (NCT03362385), executed by Beijing Anzhen Hospital, Capital Medical University, is a large-scale, prospective cohort study attempting to assess the effects exerted by OSA on cardiovascular prognosis of ACS patients. Designing schemes of the project and criteria for patient recruitment have been explicitly described in our published data [4, 5]. Briefly, 18–85 years patients admitted for ACS from June 2015 to January 2020 were recruited to undergo overnight sleep monitoring after clinical stabilization of ACS. ACS was determined when diagnosed with ST-segment elevation myocardial infarction (STEMI), non-ST-segment elevation myocardial infarction (NSTEMI), or unstable angina (UA). Patients with cardiogenic shock, cardiac arrest, previous or present utilization of continuous positive airway pressure (CPAP), malignancy (life expectancy < 2 years), and invalid recordings during sleep study were further excluded. In addition, we also precluded those with central sleep apnea (≥ 50% central events or central apnea hypopnea index (AHI) ≥ 10 events/h) prevailing, regular CPAP therapy (> 3 months) after discharge and loss of follow-up. In current study, patients with serum Hcy ≤ 15 μmol/L were defined as normohomocysteinemia (NHcy) and the other with Hcy > 15 μmol/L were classified into hyperhomocysteinemia (HHcy) group, in accordance with prior studies [25].

This study cohered with the STrengthening the Reporting of OBservational studies in Epidemiology (STROBE) guidelines, and was complied with the principles of Declaration of Helsinki. The protocol was ratified by the Ethics Committee of Beijing Anzhen Hospital, Capital Medical University (2013025). All patients were asked to sign informed consent before enrollment.

### Overnight sleep monitoring

Overnight sleep monitoring was performed by type III portable devices (ApneaLink Air, Resmed, Australia), after clinical stabilization of ACS for a median duration of 2 days. Trained research staff, blinded to the clinical characteristics of the patients, randomly assigned devices to the patients one to one before sleep and took back on the next morning to extract related data, including nasal airflow, thoracoabdominal movements, snoring episodes, heart rate, and arterial oxygen saturation. Moreover, the sleep studies were score manually according to criteria proclaimed by the American Academy of Sleep Medicine. Afterwards, apnea was defined as an absence of airflow for ≥ 10 s (coexistence of thoracoabdominal movements indicated obstructive apnea otherwise indicated central apnea), and hypopnea was identified when nasal airflow reduced by at least 30% for ≥ 10 s accompanied by arterial oxygen saturation (SaO_2_) declining > 4%. Sleep monitoring with effectiveness required sufficient polygraphy signal recording lasting for at least 3 h. Among patients undergoing successful monitoring, AHI (the sum of apneas and hypopneas per hour), oxygen desaturation index (ODI), minimum oxyhemoglobin saturation (SaO_2_), mean SaO_2_ and percentage of time with SaO_2_ < 90% (T90) were recorded. Patients with AHI ≥ 15 events/h was then diagnosed with OSA.

### Clinical procedures

During index hospitalization, patients received standard care and medications for ACS, in accordance with current guideline recommendations [26, 27]. Percutaneous coronary intervention (PCI) or coronary artery bypass grafting (CABG) was implemented if clinically indicated. Patients with AHI ≥ 15 events/h, particularly those with excessive daytime sleepiness assessed by the Epworth Sleepiness Scale (ESS) scoring, were referred to the sleep medicine center for assessment to decide further treatment, including CPAP for OSA. The baseline demographic, clinical and procedural information were comprehensively collected.

### Follow-up and endpoints

Clinical visits, which was fulfilled via clinic visit, medical records, or telephone calls by researchers blinded to the patients’ data, were scheduled at 1 month, 3 months, 6 months, 12 months, and every 6 months thereafter since index hospitalization.

The primary endpoint was major adverse cerebrocardiovascular events (MACCE) composed of cardiovascular death, recurrent myocardial infarction (MI), stroke, ischemia-driven revascularization, and hospitalization for UA and heart failure (HF). Secondary endpoints comprised every component of primary endpoint, all-cause death, all repeat revascularization, and non-culprit revascularization. All endpoints were complied with the definitions published by the Standardized Data Collection for Cardiovascular Trials Initiative [28] (Additional file 1: Methods).

### Statistical analysis

Baseline characteristics, sleep monitoring results and outcomes were stratified by Hcy level (≤ 15 μmol/L or > 15 μmol/L) and OSA status (AHI ≥ 15 events/h or < 15 events/h). Continuous variables were presented as mean ± standard deviation or median (interquartile: first and third quartiles) and were compared by the Student *t* test or Mann–Whitney *U* test, respectively. Categorical variables were shown as the number (percentage) and were compared by χ^2^ statistics or Fisher’s exact test, as appropriate.

In outcome analyses, time-to-event data and cumulative incidence of primary endpoint and secondary endpoints according to the interaction between Hcy and OSA were plotted by Kaplan–Meier curves, and the difference was examined by log-rank test. Schoenfeld residuals were checked to ensure the proportionality assumption in the Cox model for OSA status in relation to MACCE, which was confirmed by a *P* > 0.05. Univariable and multivariable Cox analyses were executed in both Hcy groups, respectively, to calculate the hazard ratios (HRs) with 95% confidence interval (CI), from which the risk of OSA patients for the occurrence of MACCE and key secondary endpoints, compared to those without OSA, was evaluated. A Finn-Gray model was executed to assess whether competing risk existed between MACCE and non-cardiogenic death. Covariates incorporated in multivariable Cox proportional hazards model were mainly based on the baseline variables which were considered clinically relevant or those showed a univariable association with primary outcomes, encompassing age, gender, body mass index (BMI), hypertension, diabetes mellitus, hyperlipidemia, prior MI, prior stroke, ACS types, smoking status, drinking and estimated glomerular filtration rate (eGFR) < 90 mL/min/1.73 m^2^. Variables for inclusion were carefully selected in consideration of the number of events available, to fulfill parsimony and accuracy of the final models.

Subgroup analyses of primary endpoint in both Hcy groups were executed based on important characteristics of interest, including age (< 65 or ≥ 65 years), gender, BMI (< 28 or ≥ 28 kg), hypertension (yes or no), diabetes mellitus (yes or no), hyperlipidemia (yes or no), ACS types (STEMI or NSTE-ACS), prior MI (yes or no), prior stroke (yes or no) and renal dysfunction (eGFR < 90 or ≥ 90 mL/min/1.73 m^2^), where the HRs were adjusted for confounders similar to outcome analyses, except for the grouping variable. Additionally, multiplicative interaction terms were appended to the adjusted COX models to assess whether the grouping variables modified the associations between OSA and risk for MACCE incidence.

All statistical analyses were conducted with SPSS (version 26.0, IBM SPSS Inc, Armonk, NY, USA) and R Statistical Software (version 4.2.0; R Foundation for Statistical Computing, Vienna, Austria). A two-tailed *P* < 0.05 was considered statistically significant.

## Results

### Study population and factors associated with Hcy level

**Fig. 1:**
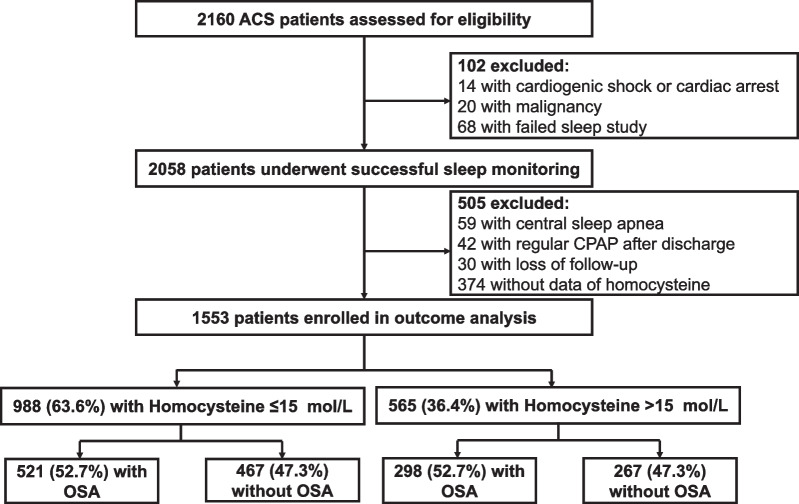
Flow diagram of patient enrollment. *ACS* acute coronary syndrome, *CPAP* continuous positive airway pressure, *OSA* obstructive sleep apnea

**Fig. 2:**
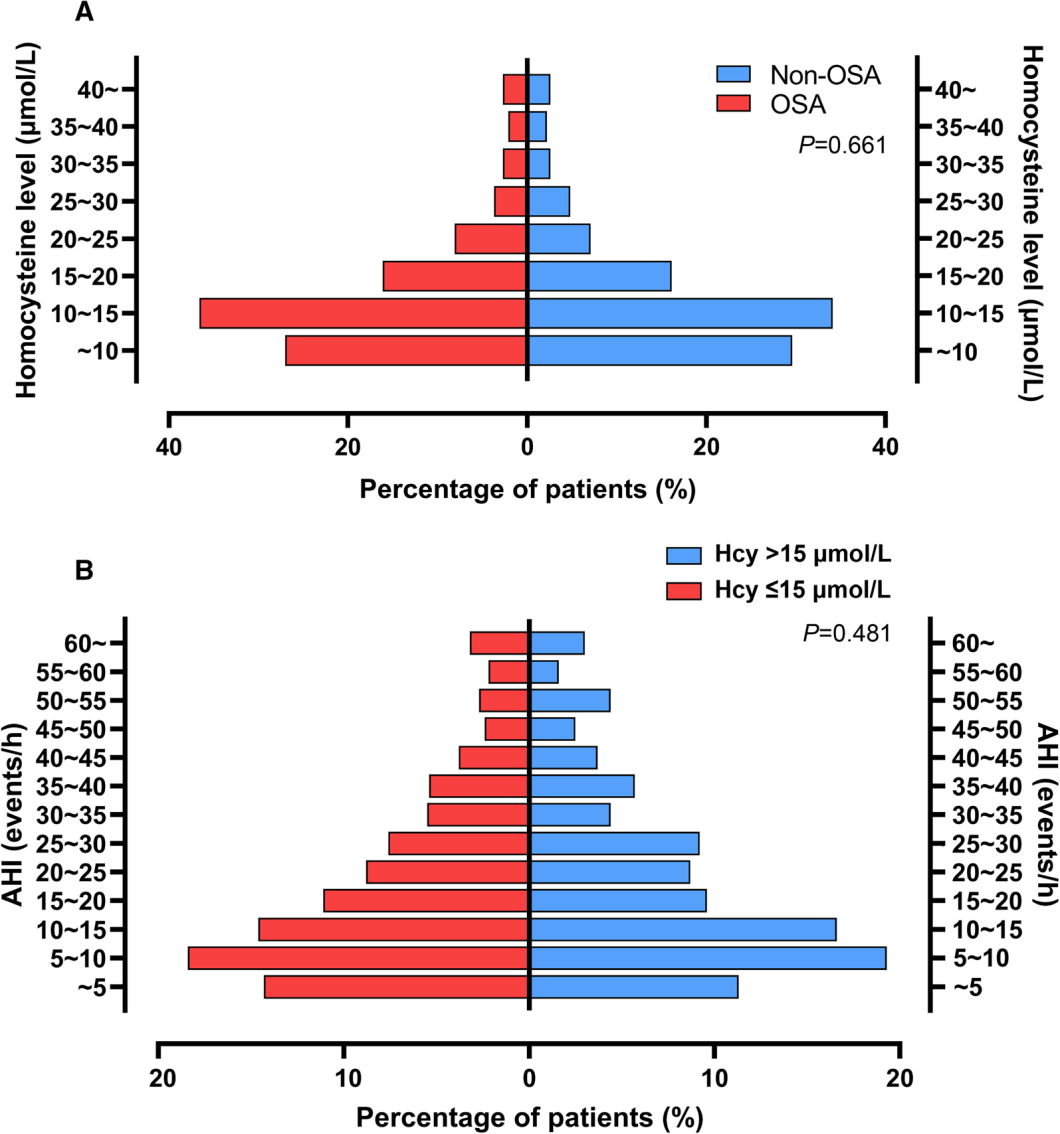
Association between homocysteine level and OSA severity in overall patients. Distribution patterns of all homocysteine subgroups between OSA and non-OSA patients (**A**); Distribution patterns of all apnea hypopnea index subgroups between patients with normal and high homocysteine (**B**). *AHI* apnea hypopnea index, *OSA* obstructive sleep apnea

A total of 2160 ACS patients were assessed for eligibility, among which 2058 underwent successful overnight sleep monitoring, followed by exclusion of patients with central sleep apnea (n = 59), regular CPAP therapy after discharge (n = 42), loss of follow-up (n = 30) and those without Hcy data (n = 374). Eventually, 1553 were involved in further analyses, with an average age of 56.3 ± 10.5 years (Fig. 1). Among them, 819 (52.7%) patients had AHI ≥ 15 events/h. We subsequently observed analogous level and distribution pattern of Hcy between OSA and non-OSA patients (13.1 [9.8, 17.9] vs. 12.8 [9.6, 18.8], *P* = 0.598; Fig. 2A, P = 0.661). Furthermore, the distribution patterns of AHI, which indicated severity of OSA, also did not differ between HHcy and NHcy patients (Fig. 2B, P = 0.481; Additional file 2: Table S1). In contrast, we identified several factors associated with the variance of Hcy in ACS patients, including advanced age, gender, diabetes mellitus, smoking and renal function (Additional file 3: Fig. S1).

### Baseline characteristics and results of overnight sleep monitoring

Nine hundred and eighty eight patients with Hcy ≤ 15 μmol/L were defined as NHcy while 565 patients with Hcy > 15 μmol/L were classified into HHcy group, among which 521 (52.7%) and 298 (52.7%) were diagnosed with OSA, respectively. Baseline characteristics stratified by the interaction between Hcy level and OSA status were summarized in Table 1. OSA Patients were more often male, obese and were more likely with hypertension versus those with relatively normal nocturnal airflow, regardless of Hcy level. Most of other variables, including medical history, medications, disease and procedural characteristics and laboratory examinations, were comparable between OSA and non-OSA patients. In addition, ACS patients with HHcy were less likely comorbid with diabetes mellitus and hyperlipidemia, but with higher rates of prior stroke, current smoker and STEMI, versus NHcy patients.

**Table 1:** Baseline characteristics

Variables	Homocysteine ≤ 15 μmol/L		*P*	Homocysteine > 15 μmol/L		*P*	P^*^
	OSA (n = 521)	Non-OSA (n = 467)		OSA (n = 298)	Non-OSA (n = 267)		
Age, years	56.6 ± 10.0	55.7 ± 10.6	0.162	56.4 ± 11.1	56.7 ± 10.4	0.774	0.476
Male, n (%)	433 (83.1)	356 (76.2)	0.007	283 (95.0)	239 (89.5)	0.015	< 0.001
BMI, kg/m^2^	27.9 ± 3.6	26.1 ± 3.4	< 0.001	28.0 ± 3.4	26.0 ± 3.5	< 0.001	0.889
Heart rate, bpm	71 (65, 80)	70 (64, 78)	0.032	70 (64, 79)	70 (64, 77)	0.672	0.064
Systolic pressure, mmHg	126 (117, 138)	127 (116, 139)	0.974	128 (117, 140)	125 (117, 136)	0.205	0.953
Diastolic pressure, mmHg	76 (70, 85)	75 (69, 83)	0.027	79 (70, 87)	76 (70, 82)	0.027	0.148
Medical history							
Prior MI, n (%)	89 (17.1)	67 (14.3)	0.239	56 (18.8)	44 (16.5)	0.472	0.329
Prior PCI, n (%)	119 (22.8)	88 (18.8)	0.123	73 (24.5)	42 (15.7)	0.010	0.780
Heart failure, n (%)	11 (2.1)	5 (1.1)	0.217	7 (2.3)	7 (2.6)	0.835	0.237
Hypertension, n (%)	353 (67.8)	285 (61.0)	0.027	207 (69.5)	152 (56.9)	0.002	0.682
Diabetes mellitus, n (%)	186 (35.7)	178 (38.1)	0.432	75 (25.2)	48 (18.0)	0.039	< 0.001
Hyperlipidemia, n (%)	173 (33.2)	166 (35.5)	0.439	87 (29.2)	63 (23.6)	0.132	0.002
Prior stroke, n (%)	55 (10.6)	29 (6.2)	0.014	39 (13.1)	36 (13.5)	0.890	0.003
Renal impairment, n (%)	7 (1.3)	8 (1.7)	0.635	9 (3.0)	6 (2.2)	0.568	0.117
Current smoking, n (%)	239 (45.9)	186 (39.8)	0.055	167 (56.0)	152 (56.9)	0.832	< 0.001
Drinking, n (%)	209 (40.1)	148 (31.7)	0.006	133 (44.6)	108 (40.4)	0.316	0.011
Family history of CAD, n (%)	22 (4.2)	30 (6.4)	0.122	20 (6.7)	9 (3.4)	0.072	0.911
Medications							
Prescription at discharge							
Aspirin, n (%)	511 (98.1)	453 (97.0)	0.272	286 (96.0)	262 (98.1)	0.135	0.493
P2Y12 inhibitor, n (%)	478 (91.7)	421 (90.1)	0.381	275 (92.3)	246 (92.1)	0.948	0.408
β-blocker, n (%)	395 (75.8)	360 (77.1)	0.638	233 (78.2)	187 (70.0)	0.027	0.358
ACEI/ARB, n (%)	338 (64.9)	273 (58.5)	0.038	200 (67.1)	158 (59.2)	0.051	0.552
Statin, n (%)	514 (98.7)	459 (98.3)	0.635	295 (99.0)	264 (98.9)	0.892	0.454
Disease characteristics							
ACS type, n (%)			0.120			0.120	0.031
STEMI	118 (22.6)	87 (18.6)		84 (28.2)	60 (22.5)		
NSTE-ACS	403 (77.4)	380 (81.4)		214 (71.8)	207 (77.5)		
Stent implantation, n (%)	292 (56.0)	222 (47.5)	0.008	172 (57.7)	147 (55.1)	0.524	0.092
CABG, n (%)	24 (4.6)	38 (8.1)	0.022	19 (6.4)	23 (8.6)	0.311	0.380
Laboratory examinations							
LVEF, %	60 (55, 65)	61.5 (56, 65)	0.013	60 (55, 65)	61 (56, 65)	0.276	0.015
Homocysteine, μmol/L	10.7 (9.1, 12.8)	10.4 (8.9, 12.4)	0.165	21.1 (16.8, 29.2)	21.0 (17.6, 29.3)	0.412	< 0.001
FPG, mmol/L	6.12 (5.39, 8.22)	6.00 (5.34, 7.57)	0.216	5.78 (5.30, 7.15)	5.61 (5.21, 6.45)	0.040	< 0.001
HbA1c, %	6.2 (5.7, 7.6)	6.1 (5.6, 7.2)	0.238	6.0 (5.6, 6.6)	5.8 (5.5, 6.5)	0.095	< 0.001
Total cholesterol, mmol/L	4.18 (3.51, 4.86)	4.09 (3.39, 4.98)	0.461	4.16 (3.51, 4.97)	4.19 (3.56, 4.98)	0.654	0.192
Triglyceride, mmol/L	1.53 (1.13, 2.25)	1.43 (1.05, 2.18)	0.035	1.57 (1.13, 2.27)	1.48 (1.06, 2.15)	0.260	0.512
LDL-C, mmol/L	2.43 (1.94, 3.06)	2.36 (1.81, 3.07)	0.177	2.46 (1.95,3.17)	2.57 (1.97, 3.25)	0.538	0.019
HDL-C, mmol/L	0.99 (0.86, 1.14)	1.03 (0.88, 1.19)	0.010	0.97 (0.86, 1.13)	1.02 (0.85, 1.17)	0.116	0.151
eGFR, mL/min/1.73 m^2^	106.8 (92.4 121.7)	110.7 (95.5, 126.0)	0.003	98.5 (81.4, 115.3)	102.4 (82.2, 118.4)	0.240	< 0.001

Data are presented as median

*IQR* first and third quartiles or number (percentage), *BMI* body mass index, *MI* myocardial infarction, *PCI* percutaneous coronary intervention, *CAD* coronary artery disease, *ACEI/ARB* Angiotensin-Converting Enzyme Inhibitor/angiotensin receptor blocker, *STEMI* ST-segment elevation myocardial infarction, *NSTE-ACS* non-ST-segment elevation acute coronary syndrome, *CABG* coronary artery bypass grafting, *LVEF* left ventricular ejection fraction, *FPG* fasting plasm glucose, *HbA1c* Glycosylated hemoglobin, *LDL-C* low-density lipoprotein cholesterol, *HDL-C* high-density lipoprotein cholesterol, *eGFR* estimated glomerular filtration rate

^*^Comparison between patients with homocysteine ≤ 15 μmol/L and those > 15 μmol/L

The results of sleep monitoring stratified by the interaction term (Hcy-OSA categories) were depicted in Table 2. Both in NHcy and HHcy groups, patients with OSA showed increased AHI, ODI and T90, and more apparent sleepy symptoms, concomitant with reduced minimum and mean SaO_2_, versus those without OSA (all* P* < 0.001).

**Table 2:** Overnight sleep monitoring results stratified by the interaction term between OSA and homocysteine

Variables	Homocysteine ≤ 15 μmol/L		*P*	Homocysteine > 15 μmol/L		*P*	P^*^
	OSA (n = 521)	Non-OSA (n = 467)		OSA (n = 298)	Non-OSA (n = 267)		
AHI, events/h	28.8 (20.7, 41.6)	7.7 (3.7, 10.6)	< 0.001	29.4 (21.4, 43.0)	7.8 (5.0, 11.0)	< 0.001	0.522
ODI, events/h	27.1 (20.0, 39.0)	8.6 (4.6, 12.0)	< 0.001	27.9 (21.2, 40.5)	8.8 (5.4, 11.9)	< 0.001	0.457
T90, %	6.0 (2.0, 16.0)	0.6 (0.0, 3.0)	< 0.001	6.4 (2.0, 14.0)	0.9 (0.1, 3.0)	< 0.001	0.640
Minimum SaO_2_, %	83 (77, 86)	87 (84, 90)	< 0.001	82 (77, 86)	87 (84, 89)	< 0.001	0.133
Mean SaO_2_, %	93 (92, 94)	95 (93, 95)	< 0.001	93 (92, 94)	94 (93, 95)	< 0.001	0.077
ESS scoring	8 (4, 12)	6 (3, 10)	0.001	8 (5, 12)	6 (3, 10)	0.002	0.677

*AHI* apnea and hypopnea index, *ESS* the Epworth Sleepiness Scale, *ODI* oxygen desaturation index, *T90* percentage of time with SaO_2_ < 90%, *SaO*_*2*_ oxyhemoglobin saturation

### Outcomes analyses between OSA and non-OSA patients stratified by Hcy level

**Fig. 3:**
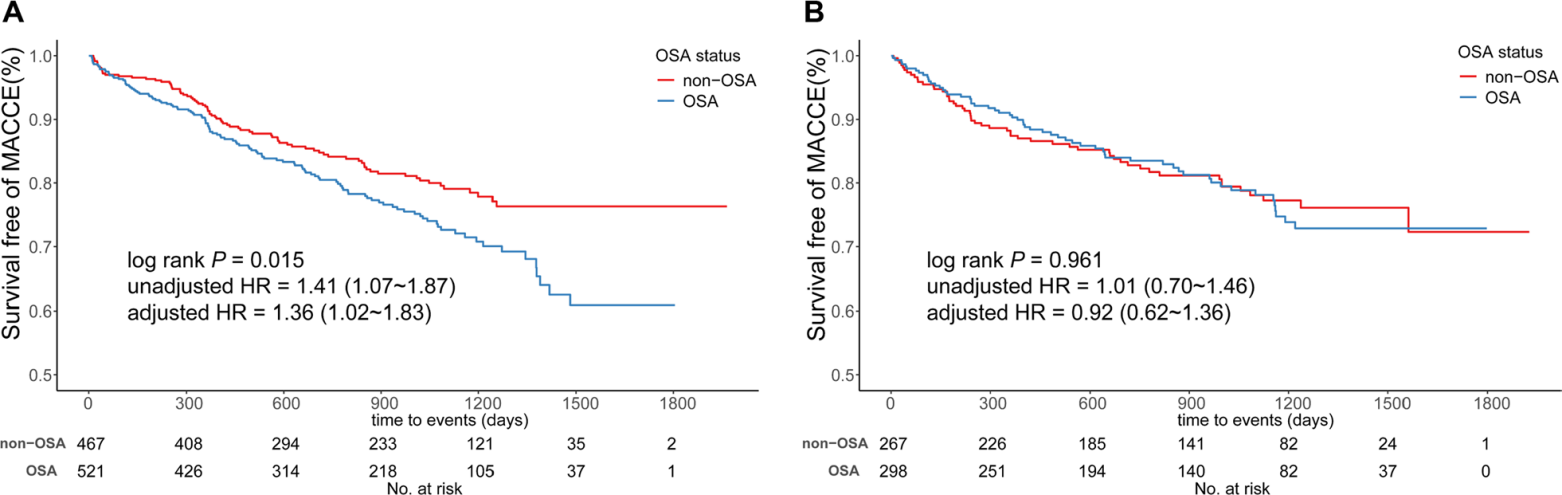
Kaplan–Meier curves for primary endpoint according to age-OSA categories. Exposure to OSA significantly increased the incidence of major adverse cerebrocardiovascular events (MACCE) in ACS patients with normohomocysteinemia (**A**), unlike in ACS patients with hyperhomocysteinemia (**B**)

During a median follow-up of 2.9 years (interquartile range: 1.6–3.5 years), 317 (20.4%) patients suffered from the recurrence of MACCE, with 180 (22.0%) in the OSA group and 137 (18.7%) in the non-OSA group, respectively. However, the incident rate of MACCE was comparable between NHcy and HHcy groups (20.6% vs. 20.0%, log rank *P* = 0.474) (Additional file 4: Fig. S2). Table 3 summarized all relevant outcome data. Hospitalization for UA and ischemia-driven revascularization accounted for the majority of MACCE. Intriguingly, we found that among ACS patients with NHcy, those who developed OSA had a significantly increased crude incidence of MACCE versus non-OSA ones (23.2% vs. 17.8%, log rank *P* = 0.015, Fig. 3A), which, in turn, was insignificant among HHcy patients (19.8% vs. 20.2%, log rank *P* = 0.961, Fig. 3B). Univariable COX analyses further verified this divergence (in NHcy patients, unadjusted HR = 1.41, 95%CI 1.07–1.87, *P* = 0.015; in HHcy patients, unadjusted HR = 1.01, 95%CI 0.70–1.46, *P* = 0.961; Table 3). Moreover, after adjustment for clinical confounders, OSA was still associated with increased risk for MACCE in ACS patients with NHcy (adjusted HR = 1.36, 95%CI 1.02–1.83, *P* = 0.039), but not in HHcy patients (adjusted HR = 0.92, 95%CI 0.62–1.36, *P* = 0.668) (Table 3). After controlling competing risk for non-cardiogenic death, OSA was still associated with long-term events in patients with normal level of homocysteine (HR = 1.36, 95%CI 1.02–1.83, P = 0.039), but not in those with hyperhomocysteinemia (HR = 0.92, 95%CI 0.62–1.36, P = 0.668). There was an absence of interaction between OSA and homocysteine with respect to MACCE (interaction *P* = 0.106).

**Table 3:** Crude incidence and COX analyses of all relevant endpoints

Clinical outcomes	Subgroups of homocysteine	OSA, n (%)	Non-OSA, n (%)	Unadjusted HR (95% CI)	Adjusted HR^a^ (95% CI)
Major adverse cerebrocardiovascular events	NHcy	121 (23.2)	83 (17.8)	1.41 (1.07–1.87)	1.36 (1.02–1.83)
	HHcy	59 (19.8)	54 (20.2)	1.01 (0.70–1.46)	0.92 (0.62–1.36)
Hospitalization for UA	NHcy	87 (16.7)	64 (13.7)	1.30 (0.94–1.79)	1.27 (0.90–1.78)
	HHcy	41 (13.8)	34 (12.7)	1.13 (0.72–1.78)	1.09 (0.67–1.77)
Cardiovascular death	NHcy	9 (1.7)	6 (1.3)	1.39 (0.50–3.91)	1.31 (0.43–3.95)
	HHcy	5 (1.7)	7 (2.6)	0.65 (0.21–2.05)	0.34 (0.09–1.26)
Recurrent MI	NHcy	13 (2.5)	12 (2.6)	1.01 (0.46–2.22)	0.86 (0.37–2.03)
	HHcy	11 (3.7)	4 (1.5)	2.51 (0.80–7.91)	2.26 (0.68–7.52)
Stroke	NHcy	13 (2.5)	6 (1.3)	2.03 (0.77–5.35)	1.92 (0.69–5.35)
	HHcy	6 (2.0)	8 (3.0)	0.70 (0.24–2.03)	0.75 (0.24–2.34)
Ischemia-driven revascularization	NHcy	50 (9.6)	37 (7.9)	1.26 (0.83–1.93)	1.15 (0.73–1.81)
	HHcy	23 (7.7)	19 (7.1)	1.12 (0.61–2.06)	1.10 (0.58–2.10)
Hospitalization for HF	NHcy	4 (0.8)	3 (0.6)	1.27 (0.28–5.66)	1.29 (0.25–6.59)
	HHcy	5 (1.7)	6 (2.2)	0.73 (0.22–2.39)	0.32 (0.08–1.29)
All repeated revascularization	NHcy	72 (13.8)	51 (10.9)	1.33 (0.93–1.91)	1.25 (0.85–1.84)
	HHcy	31 (10.4)	31 (11.6)	0.91 (0.55–1.50)	0.82 (0.49–1.40)
Non-culprit revascularization	NHcy	48 (9.2)	28 (6.0)	1.59 (1.00–2.54)	1.59 (0.97–2.61)
	HHcy	20 (6.7)	19 (7.1)	0.96 (0.51–1.79)	0.85 (0.44–1.66)
All-cause death	NHcy	11 (2.1)	11 (2.4)	0.93 (0.40–2.15)	0.89 (0.36–2.17)
	HHcy	6 (2.0)	8 (3.0)	0.68 (0.24–1.96)	0.52 (0.16–1.71)

*NHcy* normohomocysteinemia, *HHcy* hyperhomocysteinemia, *UA* unstable angina, *MI* myocardial infarction, *HF* heart failure

^a^Adjusted for age (continuous variable), gender (males or females), BMI (continuous variable), hypertension (yes or no), diabetes mellitus (yes or no), hyperlipidemia (yes or no), prior MI (yes or no), prior stroke (yes or no), ACS types (STEMI or NSTE-ACS), smoke status (current or no), drinking (yes or no) and eGFR < 90 mL/min/1.73 m^2^ (yes or no)

However, association between OSA and every secondary endpoint was not statistically significant both in NHcy and HHcy groups (Table 3). Notably, among NHcy patients, OSA nominally increased the risk for hospitalization for UA (adjusted HR = 1.27, 95%CI 0.90–1.78, *P* = 0.174) and non-culprit revascularization (adjusted HR = 1.59, 95%CI 0.97–2.61, *P* = 0.067).

### Independent risk factors of outcomes stratified by Hcy level

Independent risk factors of clinical outcomes according to Hcy level were shown in Table 4. Intriguingly, among ACS patients with NHcy, only OSA and diabetes mellitus (HR = 1.33, 95%CI 1.00–1.77, *P* = 0.048) independently predicted the occurrence of MACCE. In stark contrast, in HHcy patients, several risk factors (age, female, diabetes mellitus, prior MI, prior stroke and current smoking) showed potential association with MACCE in univariable analysis (*P* < 0.100), which, however, transformed to insignificance in multivariable models.

**Table 4:** Risk factors of MACCE stratified by level of homocysteine

Variables	Univariable		Multivariable	
	HR (95% CI)	*P*	HR (95% CI)	*P*
Patients with Homocysteine ≤ 15 μmol/L				
OSA	1.41 (1.07–1.87)	0.015	1.36 (1.02–1.83)	0.039
Age	1.01 (1.00–1.03)	0.098	1.01 (0.99–1.02)	0.402
Female	0.99 (0.71–1.38)	0.958	0.89 (0.60–1.32)	0.563
BMI	1.01 (0.98–1.05)	0.503	1.00 (0.96–1.04)	0.941
Hypertension	1.34 (0.99–1.81)	0.057	1.22 (0.89–1.67)	0.219
Diabetes mellitus	1.36 (1.03–1.80)	0.029	1.33 (1.00–1.77)	0.048
Hyperlipidemia	0.94 (0.69–1.27)	0.671	0.96 (0.70–1.32)	0.794
Prior MI	1.01 (0.70–1.47)	0.956	0.98 (0.67–1.44)	0.925
Prior stroke	1.75 (1.15–2.66)	0.009	1.50 (0.97–2.31)	0.067
STEMI	1.15 (0.84–1.58)	0.391	1.22 (0.88–1.69)	0.242
Current smoking	0.96 (0.73–1.27)	0.798	1.05 (0.76–1.44)	0.774
Drinking	0.88 (0.65–1.18)	0.387	0.88 (0.64–1.21)	0.432
eGFR < 90 mL/min/1.73 m^2^	1.39 (1.00–1.93)	0.049	1.25 (0.88–1.76)	0.206
Patients with homocysteine > 15 μmol/L				
OSA	1.01 (0.70–1.46)	0.961	0.92 (0.62–1.36)	0.668
Age	1.02 (1.01–1.04)	0.010	1.02 (1.00–1.04)	0.131
Female	1.96 (1.15–3.32)	0.013	1.46 (0.78–2.75)	0.237
BMI	1.02 (0.97–1.07)	0.436	1.04 (0.98–1.09)	0.206
Hypertension	1.37 (0.92–2.06)	0.121	1.18 (0.77–1.80)	0.448
Diabetes mellitus	1.44 (0.95–2.17)	0.086	1.16 (0.75–1.80)	0.500
Hyperlipidemia	1.24 (0.82–1.86)	0.313	1.11 (0.73–1.69)	0.632
Prior MI	1.50 (0.97–2.33)	0.071	1.45 (0.92–2.27)	0.106
Prior stroke	1.90 (1.19–3.03)	0.007	1.60 (0.98–2.62)	0.060
STEMI	1.17 (0.78–1.75)	0.440	1.43 (0.93–2.18)	0.102
Current smoking	0.72 (0.50–1.04)	0.082	0.85 (0.56–1.29)	0.441
Drinking	1.00 (0.68–1.45)	0.986	1.21 (0.81–1.83)	0.354
eGFR < 90 mL/min/1.73 m^2^	1.35 (0.93–1.97)	0.112	1.09 (0.72–1.64)	0.686

*BMI* body mass index, *MI* myocardial infarction, *STEMI* ST-segment elevation myocardial infarction, *eGFR* estimated glomerular filtration rate

### Subgroups analyses of outcomes

The association between OSA and the risk for MACCE incidence were further evaluated based on distinct subgroups, encompassing age, gender, obesity, hypertension, diabetes mellitus, hyperlipidemia, ACS types, prior MI, prior stroke and renal dysfunction (Additional file 5: Fig. S3), where none of differences were found, and the association of OSA with MACCE was not modified by these confounding factors (all *P* for interaction ≥ 0.171).

## Discussion

In current study, based on a large-scale, prospective OSA-ACS cohort, we found that the presence of OSA did not change the serum Hcy level of ACS patients, and OSA was only associated with increased risk for cardiovascular events among those with NHcy, but not in HHcy patients. Our study firstly emphasized a fact that ACS patients with a relatively normal level of Hcy also suffered from high risk for cardiovascular events, which was attributed partially to concomitant OSA. Therefore, aggressive screening, definitive diagnosis and effective interventions for OSA especially in such a population were recommended aiming to ameliorate the prognosis.

With fulfillment of great progress in secondary prevention strategies for traditional cardiovascular risk factors (e.g. hyperlipidemia, hypertension, diabetes mellitus) and in revascularization therapy, the overall morbidity, mortality and prognosis of coronary artery disease (CAD) or ACS have been improved over time [29–32]. However, in 2021, Figtree and the colleagues observed a higher short-term mortality in STEMI patients without standard modifiable cardiovascular risk factors, versus those with at least one [33], yielding the exploration of residual risk factors. Almost simultaneously, the American Heart Association appended sleep health as a novel metric of cardiovascular health [34], where OSA is an essential component, a novel and paramount risk factor involved in the pathogenesis and progression of cardiovascular diseases according to contemporary notion [3, 8].

A plethora of studies have demonstrated detrimental effects exerted by OSA on ACS patients. OSA was involved in the exacerbation of ACS, evidenced by increased cardiac injury, and plaque burden and vulnerability, which were responsible for worse cardiovascular prognosis [35–37]. In the Sleep and Stent Study, exposure to OSA increased the risk for the occurrence of MACCE by 57% in CAD patients undergoing PCI, among which 68.5% were ACS, during a median follow-up of 1.9 years [38]. Subsequently, in the mid-term analysis of OSA-ACS project containing 804 ACS patients [4], we reported approximately fourfold risk for the incidence of MACCE in those comorbid with OSA after 1-year follow-up. CPAP has been recommended as the first-line treatment for OSA. CPAP treatment was associated with mild-to-moderate reduction of blood pressure, alleviated sleepy symptoms, improvement of cardiac function and arrhythmias in ACS patients [6, 9, 39], which could contribute synergistically to improving the prognosis [40]. However, in recent, ISAACC studies [6], the largest-scale randomized controlled trials focusing on the therapeutic efficacy of CPAP on ACS patients, demonstrated that CPAP treatment failed to protect against long-term cardiovascular events or sleepy symptoms. Although inclusion of non-sleepy patients and suboptimal adherence might be partially responsible for the negative results, there was a hypothesis that patients with high-risk clinical phenotypes might respond better to CPAP treatment [41]. We have previously identified several specific populations vulnerable to OSA, comprising diabetes mellitus, females, non-obesity and hyperuricemia [5, 11, 12, 42], with the impact stratified by Hcy remaining unelucidated.

Cardiometabolic dysregulation played a crucial role on this association with respect to the fact that OSA interacted diabetes mellitus, dyslipidemia and hypertension with reciprocal causation [43–45]. One of these metabolites by rational speculation was Hcy due to the common mechanisms underlying cardiovascular injuries between Hcy and OSA. Nevertheless, the association between OSA and Hcy was controversial. Numerous investigators observed an elevation of Hcy level in OSA patients with or without prior cardiovascular diseases [19, 20, 46–48]. Conversely, Svatikova et al. executed a rigorously controlled study enrolling obese but otherwise healthy patients where Hcy level was comparable between OSA and control subjects and neither nocturnal OSA nor sleep disturbance transiently changed Hcy [14]. In addition, CPAP treatment appeared not to affect Hcy [21, 49], further supporting the notion that OSA itself was not associated with the level of Hcy. However, OSA patients were more likely to be elder, male, obese, and concomitant with renal dysfunction, which were prominent risk factors of Hcy elevation [16]. In our current study, we also found that in ACS patients, OSA did not modify the level of Hcy, which, in turn, varied with age, gender, diabetes mellitus, renal dysfunction and smoking. Therefore, coexistence of OSA and elevated Hcy might reflect several variances in metabolic conditions, the identification of which required the implementation of trials with larger sample size and rigorous schemes.

It was noticeable that the effects of Hcy on cardiovascular outcomes among ACS patients were also disputed. A plethora of studies have identified elevated Hcy as a strong independent risk factor of worse cardiovascular outcomes in ACS patients [17, 50]. However, Foussas et al. reported an absence of association of between Hcy and long-term mortality in neither STEMI nor NSTE-ACS cohort, after adjustment for prominent risk factors and medical history [22]. A rational explanation for this controversy might be the heterogeneity of recruited patients, adjusted confounders included in multivariable models and a relatively small sample size [22]. Therefore, in our study by utilization of 1553 ACS patients, we observed a comparable incident rate of MACCE between NHcy and HHcy groups. Another convictive evidence against the role for Hcy was that folic acid and vitamin B_6_/B_12_ supplement, aiming to prompt the metabolic consumption of Hcy, failed to ameliorate the cardiovascular outcomes of acute myocardial infarction patients in spite of substantial reduction of Hcy [51]. Thus, Hcy might be rather a biomarker clustered with risk factor profiles, than a modifiable risk factor [22]. In addition, although synergistical impacts of OSA and augmented Hcy on cardiovascular events in patients without established ACS was observed [23, 24], there was a knowledge gap of this interaction among ACS patients. However, in HHcy patients, OSA did not increase the risk for MACCE versus non-OSA ones. In stark contrast, ACS patients with NHcy were more vulnerable to the jeopardies derived from OSA. Furthermore, we adjusted several important confounders responsible for Hcy level to avoid potential impacts, including age, gender, renal function, diabetes mellitus and current smoking, and OSA still independently predicted the incidence of MACCE. Taken together, we reasonably hypothesized that comorbid condition of OSA with HHcy might exert a ceiling effect on cardiovascular injuries, where the roles for OSA were overwhelmed, by virtue of that various risk factors often concomitant with OSA showed potential association with MACCE in these patients. Another explanation was that OSA patients with metabolic dysfunction were recommended to have more aggressive lifestyle improvements, including diet, exercise and the combination [3, 52, 53]. A meta-analysis based on randomized controlled trials indicated that weight loss of 14 kg was associated with a reduction of AHI of 16 evens/h and a part of patients could even achieve remission of OSA [54]. Further studies should take lifestyle interventions into account to distinguish the effects of OSA during long-term follow-up. Our study emphasized that the risk for long-term MACCE in ACS patients with NHcy should not be neglected, which might be attributed partially to concomitant OSA. Therefore, it was necessary to execute aggressive screening and diagnosis for OSA, favorable for individual risk stratification and therapy guiding, in such a population. This was of paramount importance to ameliorate cardiovascular outcomes since OSA was prevalent and treatable, and further studies were also required to investigate whether CPAP treatment in ACS patients with NHcy was effective.

## Limitations

There were several limitations in our study. First, the OSA-ACS project is a single-center, observational study with inevitable confounding bias. Thus, to control this limitation, professional researchers collecting follow-up data were blinded to the patients’ sleep results and baseline characteristics. Moreover, we adjusted for several important confounders in the outcome analyses to ensure the reliability of results, to an extent. Second, the definition of OSA according to portable sleep monitors may underestimate the severity of OSA. However, portable polygraphy has been validated to substitute for polysomnography with an effectiveness in previous studies [55]. Third, the data from formal sleep center follow-up visits and OSA treatment adherence after discharge was not included in current study, the effects of OSA treatment on MACCE could not be assessed in this study. Fourth, we did not take the effects of lifestyle improvements into account during follow-up. Finally, our study focused on East Asian patients, whether similar findings could extend to other ethnicities required further investigation.

## Conclusion

In our current study, OSA did not significantly affect the level of Hcy in ACS patients. Furthermore, Hcy level-dependent effects of OSA on cardiovascular outcomes were observed, where OSA was associated with increased risk for MACCE during long-term follow-up in ACS patients with relatively normal Hcy, but not in those with elevated Hcy. Our study emphasized that regular screening, definitive diagnosis and aggressive treatment were recommended in such a population.

### Supplementary Information

**Additional file 1: Methods.** Definition of study endpoints.

**Additional file 2: Table S1.** Baseline characteristics between patients with homocysteine ≤ 15 μmol/L and those > 15 μmol/L.

**Additional file 3: Figure S1.** Factors associated with the level of homocysteine in ACS patients, comprising gender (A), diabetes mellitus (B), renal dysfunction (eGFR < 90 or not) (C), age (≥ 65 or not) (D), obesity (BMI ≥ 28 or not) (E), hypertension (F), current smoking (G) and ACS types (STEMI or NSTE-ACS) (H). ACS: acute coronary syndrome; eGFR: estimated glomerular filtration rate; BMI: body mass index; STEMI: ST-segment elevation myocardial infarction; NSTE-ACS: non-ST-segment elevation acute coronary syndrome.

**Additional file 4: Figure S2.** Kaplan–Meier curves for primary endpoint according to Hcy level.

**Additional file 5: Figure S3.** Subgroup analyses for the association between OSA and risk for incidence of MACCE. *: All HRs were adjusted for age, gender, body mass index (BMI), hypertension, diabetes mellitus, hyperlipidemia, prior MI, prior stroke, ACS types, smoking status, drinking and estimated glomerular filtration rate (eGFR) < 90 mL/min/1.73 m^2^, except for the grouping variable.

## Data Availability

All of the individual patient data collected during the study will be shared. All available data can be obtained by contacting the corresponding author (Shaoping Nie, spnie@ccmu.edu.cn). It will be necessary to provide a detailed protocol for the proposed study, to provide the approval of an ethics committee, to supply a signed data access agreement, and to have discussion with the original authors for re-analysis.

## Abbreviations

ACS: Acute coronary syndrome
AHI: Apnea hypopnea index
BMI: Body mass index
CABG: Coronary artery bypass grafting
CAD: Coronary artery disease
CPAP: Continuous positive airway pressure
eGFR: Estimated glomerular filtration rate
Hcy: Homocysteine
HHcy: Hyperhomocysteinemia
MACCE: Major adverse cerebrocardiovascular events
MI: Myocardial infarction
NHcy: Normohomocysteinemia
NSTE-ACS: Non-ST-segment elevation acute coronary syndrome
NSTEMI: Non-ST-segment elevation myocardial infarction
OSA: Obstructive sleep apnea
PCI: Percutaneous coronary intervention
STEMI: ST-segment elevation myocardial infarction
