# Supplementary material for: Prognostic implications of obstructive sleep apnea in patients with acute coronary syndrome stratified by homocysteine level: a prospective cohort study
Source: Respir Res. 2023 Dec 14;24:313. doi: 10.1186/s12931-023-02627-8 (PMC10722678; doi:10.1186/s12931-023-02627-8)
Supplement: Supplementary file 1 — Additional file 1: Methods. Definition of study endpoints. [file 12931_2023_2627_MOESM1_ESM.docx]

**Additional file 1: Methods**

**Definition of study endpoints**

**Cardiovascular death**

Death attributed to proximate cardiovascular etiologies (e.g., acute myocardial infarction, sudden cardiac arrest, heart failure, cardiogenic shock, stroke, cardiovascular hemorrhage, pulmonary embolism, aortic aneurysm rupture, dissecting aneurysm or other causes), cardiovascular procedure, or any death unless an unequivocal non-cardiovascular cause.

**Myocardial infarction**

Myocardial infarction with or without ST-segment elevation is defined according to the “Third Universal Definition of Myocardial Infarction”.

Any one of the following criteria meets the diagnosis of myocardial infarction:

- Detection of a rise and/or fall of cardiac biomarker values (preferably cardiac troponin) with at least one value above the 99th percentile upper reference limit and concomitant with at least one of the following:
- Symptoms of ischemia
- (Presumed) new significant ST-T wave changes or new LBBB
- Development of pathological Q waves
- Imaging evidence of new loss of viable myocardium or new regional wall motion abnormality
- Identification of an intracoronary thrombus by angiography or autopsy
- Cardiac death with symptoms suggestive of myocardial infarction and presumed new ischemic ECG changes or new LBBB, but death occurred before cardiac biomarkers were obtained, or before cardiac biomarker values would be increased

**Stroke**

Stroke is defined as an acute symptomatic episode of neurological dysfunction, more than 24 hours in duration in the absence of therapeutic intervention or death, due to cerebral, spinal or retinal tissue injury as evidenced by neuroimaging or lumbar puncture. It is stratified as following:

- Ischemic stroke
- Intracerebral hemorrhage
- Stroke of undetermined etiology

**Ischemia-driven revascularization**

Ischemia-driven revascularization was defined as any repeat PCI or CABG performed for either: myocardial infarction, unstable angina, stable angina, or documented silent ischemia. Repeat revascularization was further classified into target vessel or non-target vessel revascularization as well as PCI or CABG.
