# Supplementary material for: Prognostic implications of obstructive sleep apnea in patients with acute coronary syndrome stratified by homocysteine level: a prospective cohort study
Source: Respir Res. 2023 Dec 14;24:313. doi: 10.1186/s12931-023-02627-8 (PMC10722678; doi:10.1186/s12931-023-02627-8)
Supplement: Supplementary file 2 — Additional file 2: Table S1. Baseline characteristics between patients with homocysteine ≤ 15 μmol/L and those > 15 μmol/L. [file 12931_2023_2627_MOESM2_ESM.docx]

**Additional file Table S1.** Baseline characteristics between patients with homocysteine ≤15 μmol/L and those >15 μmol/L.

| Variables | Homocysteine ≤15 μmol/L (n=988) | Homocysteine > 15 μmol/L (n=565) | *P* |
| --- | --- | --- | --- |
| Age, years | 56.2±10.3 | 56.6±10.7 | 0.476 |
| Male, n (%) | 789 (79.9) | 522 (92.4) | <0.001 |
| BMI, kg/m^2^ | 27.1±3.6 | 27.1±3.6 | 0.889 |
| Heart rate, bpm | 70 (64, 80) | 70 (64, 78) | 0.064 |
| Systolic pressure, mmHg | 126 (117, 138) | 126 (117, 138) | 0.953 |
| Diastolic pressure, mmHg | 75 (70, 85) | 77 (70, 86) | 0.148 |
| **Medical history** |  |  |  |
| Prior MI, n (%) | 156 (15.8) | 100 (17.7) | 0.329 |
| Prior PCI, n (%) | 207 (21.0) | 115 (20.4) | 0.780 |
| Heart failure, n (%) | 16 (1.6) | 14 (2.5) | 0.237 |
| Hypertension, n (%) | 638 (64.6) | 359 (63.5) | 0.682 |
| Diabetes mellitus, n (%) | 364 (36.8) | 123 (21.8) | <0.001 |
| Hyperlipidemia, n (%) | 339 (34.3) | 150 (26.5) | 0.002 |
| Prior stroke, n (%) | 84 (8.5) | 75 (13.3) | 0.003 |
| Renal impairment, n (%) | 15 (1.5) | 15 (2.7) | 0.117 |
| Current Smoking, n (%) | 425 (43.0) | 319 (56.5) | <0.001 |
| Drinking, n (%) | 357 (36.1) | 241 (42.7) | 0.011 |
| Family history of CAD, n (%) | 52 (5.3) | 29 (5.1) | 0.911 |
| **Medications** |  |  |  |
| **Prescription at discharge** |  |  |  |
| Aspirin, n (%) | 964 (97.6) | 548 (97.0) | 0.493 |
| P2Y12 inhibitor, n (%) | 899 (91.0) | 521 (92.2) | 0.408 |
| β-blocker, n (%) | 755 (76.4) | 420 (74.3) | 0.358 |
| ACEI/ARB, n (%) | 611 (61.8) | 358 (63.4) | 0.552 |
| Statin, n (%) | 973 (98.5) | 559 (98.9) | 0.454 |
| **Disease characteristics** |  |  |  |
| ACS type, n (%) |  |  | 0.031 |
| STEMI | 205 (20.7) | 144 (25.5) |  |
| NSTE-ACS | 783 (79.3) | 421 (74.5) |  |
| Stent implantation, n (%) | 514 (52.0) | 319 (56.5) | 0.092 |
| CABG, n (%) | 62 (6.3) | 42 (7.4) | 0.380 |
| **Laboratory examinations** |  |  |  |
| LVEF, % | 62 (56, 66) | 61 (55, 65) | 0.015 |
| Homocysteine, μmol/L | 10.6 (9.0, 12.6) | 21.1 (17.1, 29.3) | <0.001 |
| FPG, mmol/L | 6.07 (5.36, 7.80) | 5.70 (5.24, 6.75) | <0.001 |
| HbA1c, % | 6.2 (5.7, 7.3) | 5.9 (5.6, 6.6) | <0.001 |
| Total cholesterol, mmol/L | 4.15 (3.43, 4.89) | 4.17 (3.54, 4.98) | 0.192 |
| Triglyceride, mmol/L | 1.50 (1.10, 2.21) | 1.52 (1.10, 2.23) | 0.512 |
| LDL-C, mmol/L | 2.39 (1.87, 3.06) | 2.52 (1.96,3.20) | 0.019 |
| HDL-C, mmol/L | 1.01 (0.87, 1.17) | 0.98 (0.86, 1.15) | 0.151 |
| eGFR, mL/min/1.73 m^2^ | 108.4 (93.6 123.5) | 100.1 (81.6, 116.1) | <0.001 |
| **Sleep monitoring** |  |  |  |
| AHI | 16.1 (7.9, 30.4) | 15.8 (8.2, 30.9) | 0.522 |
| ODI | 16.0 (8.7, 28.1) | 16.5 (9.1, 28.8) | 0.457 |
| T90, % | 2.3 (0.3, 10.0) | 2.6 (0.4, 9.0) | 0.640 |
| Minimum SaO_2_, % | 85 (81, 88) | 85 (80, 88) | 0.133 |
| Mean SaO_2_, % | 94 (93, 95) | 94 (93, 95) | 0.077 |

Data are presented as median (IQR: first and third quartiles) or number (percentage). BMI: body mass index; MI: myocardial infarction; PCI, percutaneous coronary intervention; CAD: coronary artery disease; ACEI/ARB: Angiotensin-Converting Enzyme Inhibitor/angiotensin receptor blocker; STEMI: ST-segment elevation myocardial infarction; NSTE-ACS, non-ST-segment elevation acute coronary syndrome; CABG: coronary artery bypass grafting; LVEF: left ventricular ejection fraction; FPG: fasting plasm glucose; HbA1c: Glycosylated hemoglobin; LDL-C: low-density lipoprotein cholesterol; HDL-C: high-density lipoprotein cholesterol; eGFR: estimated glomerular filtration rate. AHI: apnea and hypopnea index; ODI: oxygen desaturation index; T90: percentage of time with SaO2<90%; SaO2: oxyhemoglobin saturation.
