# Supplementary material for: Prognostic implications of obstructive sleep apnea in patients with acute coronary syndrome stratified by homocysteine level: a prospective cohort study
Source: Respir Res. 2023 Dec 14;24:313. doi: 10.1186/s12931-023-02627-8 (PMC10722678; doi:10.1186/s12931-023-02627-8)
Supplement: Supplementary file 3 — Additional file 3: Figure S1. Factors associated with the level of homocysteine in ACS patients, comprising gender (A), diabetes mellitus (B), renal dysfunction (eGFR < 90 or not) (C), age (≥ 65 or not) (D), obesity (BMI ≥ 28 or not) (E), hypertension (F), current smoking (G) and ACS types (STEMI or NSTE-ACS) (H). ACS: acute coronary syndrome; eGFR: estimated glomerular filtration rate; BMI: body mass index; STEMI: ST-segment elevation myocardial infarction; NSTE-ACS: non-ST-segment elevation acute coronary syndrome. [file 12931_2023_2627_MOESM3_ESM.pptx]

## Slide 1
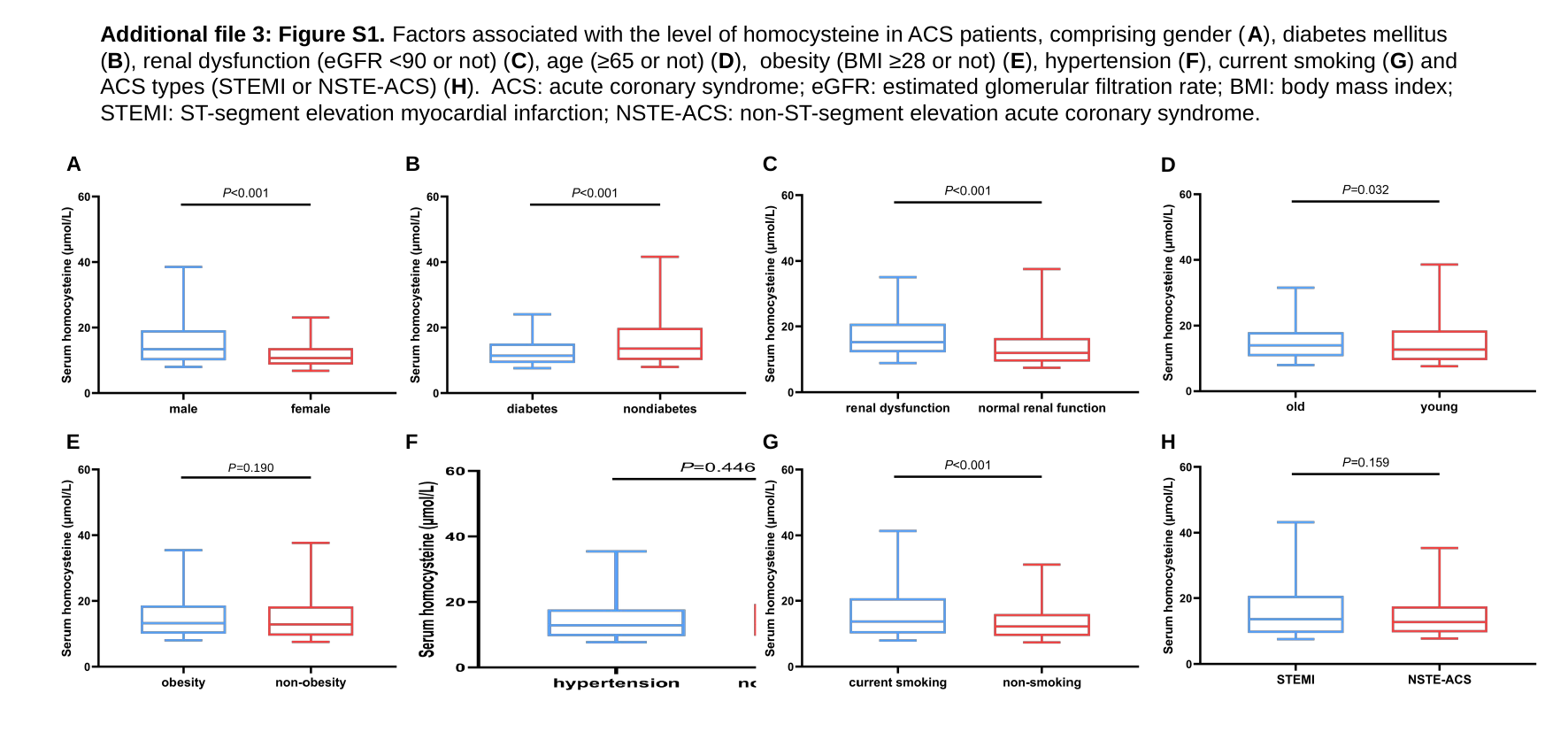

Additional file 3: Figure S1. Factors associated with the level of homocysteine in ACS patients, comprising gender (A), diabetes mellitus (B), renal dysfunction (eGFR <90 or not) (C), age (≥65 or not) (D), obesity (BMI ≥28 or not) (E), hypertension (F), current smoking (G) and ACS types (STEMI or NSTE-ACS) (H). ACS: acute coronary syndrome; eGFR: estimated glomerular filtration rate; BMI: body mass index; STEMI: ST-segment elevation myocardial infarction; NSTE-ACS: non-ST-segment elevation acute coronary syndrome.
A
B
C
D
P=0.190
H
E
F
G
