# Supplementary material for: Prognostic implications of obstructive sleep apnea in patients with acute coronary syndrome stratified by homocysteine level: a prospective cohort study
Source: Respir Res. 2023 Dec 14;24:313. doi: 10.1186/s12931-023-02627-8 (PMC10722678; doi:10.1186/s12931-023-02627-8)
Supplement: Supplementary file 4 — Additional file 4: Figure S2. Kaplan–Meier curves for primary endpoint according to Hcy level. [file 12931_2023_2627_MOESM4_ESM.pptx]

## Slide 1
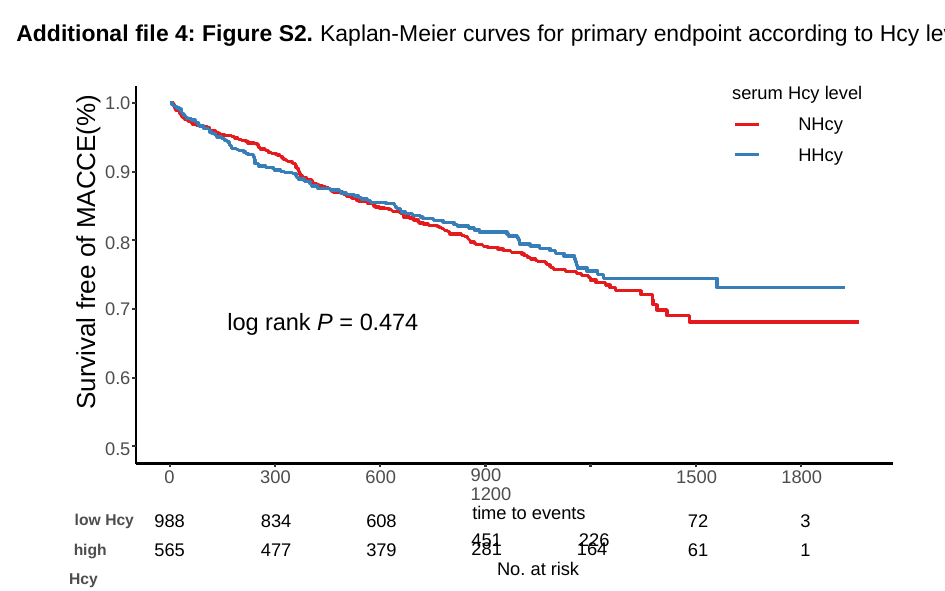

Additional file 4: Figure S2. Kaplan-Meier curves for primary endpoint according to Hcy level.
serum Hcy level NHcy HHcy
1.0
Survival free of MACCE(%)
0.9
0.8
0.7
log rank P = 0.474
0.6
0.5
900	1200
time to events
451	226
0
300
600
1500
1800
low Hcy high Hcy
988
565
834
477
608
379
72
61
3
1
281	164
No. at risk
