# Supplementary material for: Prognostic implications of obstructive sleep apnea in patients with acute coronary syndrome stratified by homocysteine level: a prospective cohort study
Source: Respir Res. 2023 Dec 14;24:313. doi: 10.1186/s12931-023-02627-8 (PMC10722678; doi:10.1186/s12931-023-02627-8)
Supplement: Supplementary file 5 — Additional file 5: Figure S3. Subgroup analyses for the association between OSA and risk for incidence of MACCE. *: All HRs were adjusted for age, gender, body mass index (BMI), hypertension, diabetes mellitus, hyperlipidemia, prior MI, prior stroke, ACS types, smoking status, drinking and estimated glomerular filtration rate (eGFR) < 90 mL/min/1.73 m2, except for the grouping variable. [file 12931_2023_2627_MOESM5_ESM.pptx]

## Slide 1
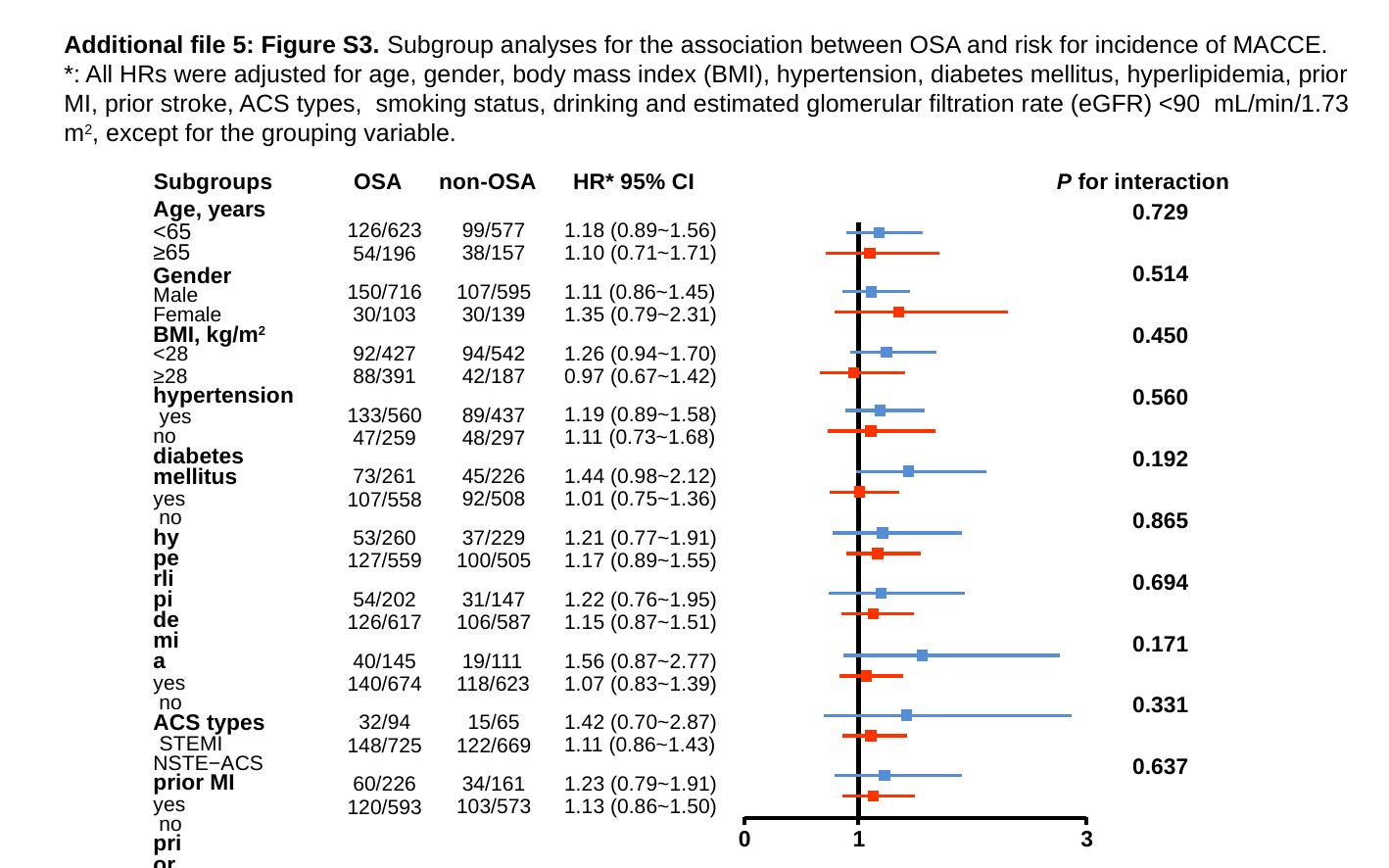

Additional file 5: Figure S3. Subgroup analyses for the association between OSA and risk for incidence of MACCE. *: All HRs were adjusted for age, gender, body mass index (BMI), hypertension, diabetes mellitus, hyperlipidemia, prior MI, prior stroke, ACS types, smoking status, drinking and estimated glomerular filtration rate (eGFR) <90 mL/min/1.73 m2, except for the grouping variable.
Subgroups OSA non-OSA HR* 95% CI P for interaction
Age, years
0.729
1.18 (0.89~1.56)
1.10 (0.71~1.71)
1.11 (0.86~1.45)
1.35 (0.79~2.31)
1.26 (0.94~1.70)
0.97 (0.67~1.42)
1.19 (0.89~1.58)
1.11 (0.73~1.68)
1.44 (0.98~2.12)
1.01 (0.75~1.36)
1.21 (0.77~1.91)
1.17 (0.89~1.55)
1.22 (0.76~1.95)
1.15 (0.87~1.51)
1.56 (0.87~2.77)
1.07 (0.83~1.39)
1.42 (0.70~2.87)
1.11 (0.86~1.43)
1.23 (0.79~1.91)
1.13 (0.86~1.50)
99/577
38/157
107/595
30/139
94/542
42/187
89/437
48/297
45/226
92/508
37/229
100/505
31/147
106/587
19/111
118/623
15/65
122/669
34/161
103/573
126/623
54/196
150/716
30/103
92/427
88/391
133/560
47/259
73/261
107/558
53/260
127/559
54/202
126/617
40/145
140/674
32/94
148/725
60/226
120/593
<65
≥65
Gender Male Female BMI, kg/m2
<28
≥28 hypertension yes
no
diabetes mellitus
yes no
hyperlipidemia
yes no
ACS types STEMI NSTE−ACS
prior MI
yes no
prior stroke
yes no
renal dysfunction
yes no
0.514
0.450
0.560
0.192
0.865
0.694
0.171
0.331
0.637
0
1
3
